# Clinical characteristics of *Chlamydia psittaci* pneumonia and predictors analysis of severe patients: a retrospective observational study

**DOI:** 10.3389/fmed.2025.1565254

**Published:** 2025-04-01

**Authors:** Ling Wu, Liang Chen, Liping Peng, Chun Liu, Shengyang He, Lihua Xie

**Affiliations:** ^1^Department of General Medicine, The Yuelushan Area of Hunan Provincial People’s Hospital, Changsha, China; ^2^Department of Radiology, The Third Xiangya Hospital of Central South University, Changsha, China; ^3^Department of Cardiology, The Third Xiangya Hospital of Central South University, Changsha, China; ^4^Department of Respiratory and Critical Care Medicine, The Third Xiangya Hospital of Central South University, Changsha, China; ^5^Department of Respiratory and Critical Care Medicine, The Second Xiangya Hospital of Central South University, Changsha, China

**Keywords:** *Chlamydia psittaci* pneumonia, community-acquired pneumonia, clinical characteristics, predictors, severe pneumonia

## Abstract

**Background:**

With this study, we aimed to explore the clinical features, laboratory examinations, imaging features, and severe predictors of *Chlamydia psittaci* pneumonia to identify the disease early, shorten the course of illness, and improve prognosis.

**Methods:**

We retrospectively reviewed the clinical data of 39 patients diagnosed with *Chlamydia psittaci* pneumonia and 39 patients with non-psittacosis community-acquired pneumonia at the Third Xiangya Hospital of Central South University from December 2018 to April 2021. We collected the remaining medical serum to analyze cytokines that are associated with disease-related inflammation. We used the R software to perform statistical analysis.

**Results:**

Compared to the non-psittacosis community-acquired pneumonia group, the common *Chlamydia psittaci* pneumonia group exhibited more severe symptoms, including a longer duration of hyperthermia. Most patients experienced dyspnea, as well as extrapulmonary symptoms such as fatigue, muscle soreness, and diarrhea. There were also significant increases in levels of alanine aminotransferase (ALT), aspartate aminotransferase (AST), lactate dehydrogenase (LDH), C-reactive protein, and procalcitonin, while hemoglobin (Hb) and albumin (ALB) levels decreased significantly. Primary lung imaging features included consolidation and exudation, with nodules and cavities being rare. These changes were even more severe in the severe *Chlamydia psittaci* pneumonia group, with further increased levels of myoglobin and a larger spread of lesions in the lungs. Additionally, the Th1 inflammatory factor INF-γ was elevated in the *Chlamydia psittaci* pneumonia group.

**Conclusion:**

Fatigue, myalgia, low Hb, low ALB, high ALT, and high AST are predictors of *Chlamydia psittaci* pneumonia. Fast respiratory rates, low Hb, high LDH, significant involvement of multiple lobes, high Sequential Organ Failure scores, and high Acute Physiological and Chronic Health scores are predictors of severe *Chlamydia psittaci* pneumonia. The increase of INF-γ may be related to the condition.

## Background

Chlamydial pneumonia is a zoonotic infection caused by *Chlamydia psittaci*, which has been reported in sporadic cases worldwide (1–5). This prokaryotic intracellular bacterium was first identified in parrots, its most common host, and can infect birds, poultry, and humans through contact with or inhalation of infected birds’ excretions (6). The clinical symptoms of this infection are non-specific and can vary (7–11). Psittacosis pneumonia, a type of community-acquired pneumonia (CAP), is often difficult to distinguish from other types of pneumonia. Currently, a definitive diagnosis of this disease requires identifying its underlying cause. However, traditional methods such as culture and serologic antibody testing can be challenging and may delay early diagnosis. This can lead to rapid progression to critical illness or even death (12). Fortunately, new detection technology, such as metagenomic next-generation sequencing (mNGS), allows for the identification of pathogens in bronchoalveolar lavage fluid (BALF) (13, 14). In fact, in our study, mNGS successfully diagnosed all confirmed cases of *Chlamydia psittaci* pneumonia (15). However, mNGS is expensive and requires a high level of skill, making it difficult to implement in most primary hospitals (16). Therefore, it is crucial to understand the clinical characteristics of *Chlamydia psittaci* pneumonia. Currently, there are inadequate researches on potential peripheral blood biomarkers for this disease (17). Previous studies have shown that *Chlamydia psittaci* infection can activate Th1 cells, which are responsible for immune responses against intracellular microorganisms. Further research is needed to determine if Th1 cell-related cytokines, such as interferon-γ (INF-γ), interleukin-2 (IL-2), and interleukin-12 (IL-12), could serve as biomarkers for this disease. Thus, the objective of this study was to describe the clinical characteristics of psittacosis pneumonia and identify potential predictors for critically ill patients. Additionally, we aimed to identify possible peripheral blood biomarkers. Improving our understanding of this disease and identifying it early can greatly improve prognoses.

## Methods

### Study design

**Figure 1:**
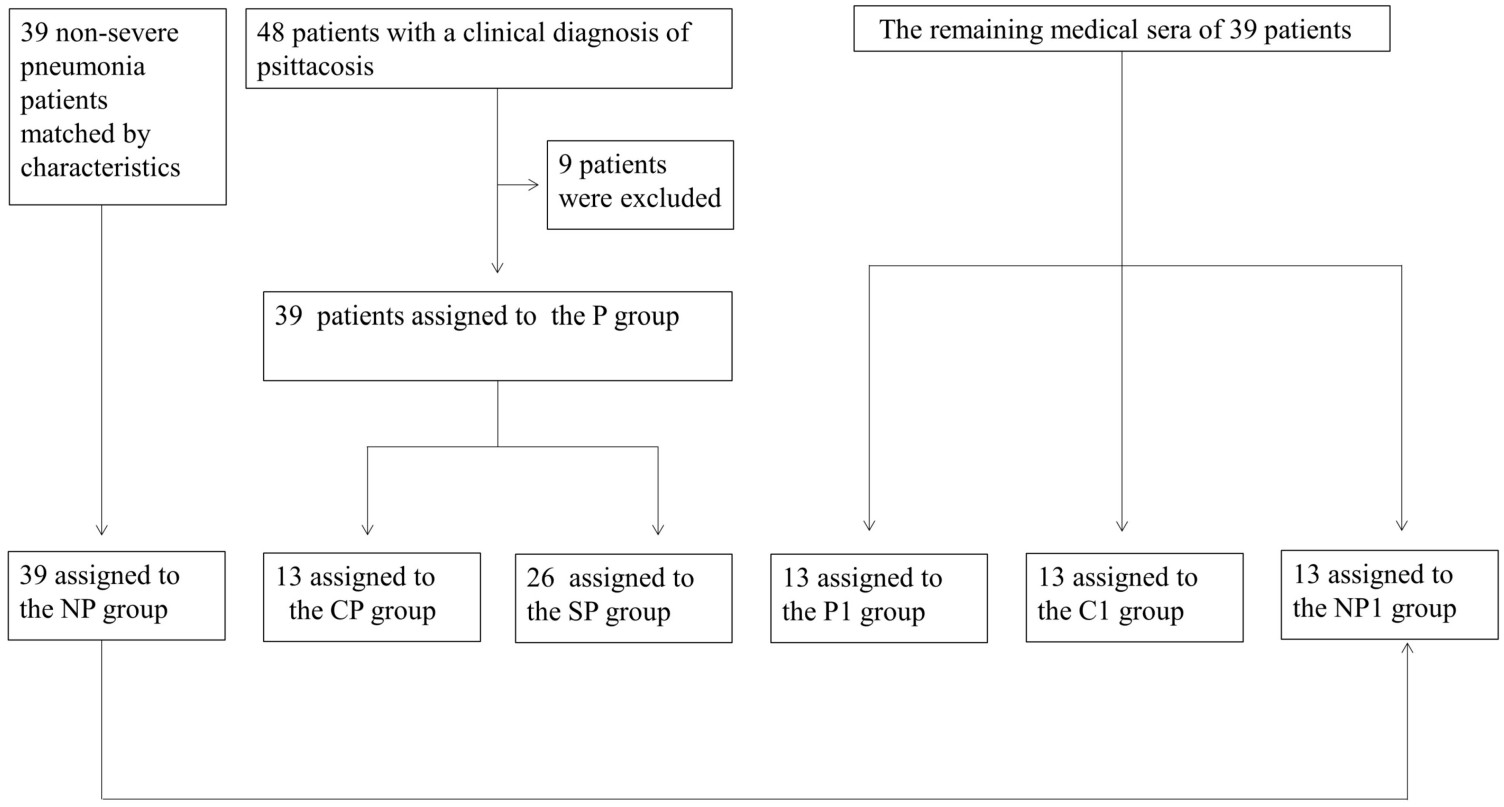
Flow diagram of the study.

We collected data from December 2018 to April 2021 on 48 patients diagnosed with *Chlamydia psittaci* pneumonia at the Third Xiangya Hospital of Central South University. Nine patients were excluded, leaving a total of 39 cases in the *Chlamydia psittaci* pneumonia group (P group). This group was further divided into 13 patients with common *Chlamydia psittaci* pneumonia (CP group) and 26 patients with severe *Chlamydia psittaci* pneumonia (SP group). Additionally, 39 non-severe pneumonia patients were matched based on characteristics and assigned to the non-psittacosis community-acquired pneumonia group (NP group). The remaining medical sera from some patients were collected prospectively and divided into three groups: 13 cases in the *Chlamydia psittaci* pneumonia sera group (P1 group), 13 cases in the healthy control sera group (C1 group), and 13 cases in the non-psittacosis community-acquired pneumonia sera group (NP1 group; Figure 1). The study was approved by the ethics committee of the Third Xiangya Hospital of Central South University (Fast I 21036), registered with the Chinese Clinical Trials Registry (ChiCTR2100045952), and conducted in accordance with the Helsinki Declaration of 1964 and its later amendments.

### Patient inclusion and exclusion criteria

#### Inclusion criteria

(1) All cases met the diagnostic and treatment guidelines for CAP in adults set by the Chinese Medical Association (18). (2) Patients with *Chlamydia psittaci* pneumonia were diagnosed by metagenomic next-generation sequencing (mNGS). (3) Severe cases of *Chlamydia psittaci* pneumonia met the above diagnostic criteria for severe pneumonia.

#### Exclusion criteria

(1) Cases with incomplete medical records. (2) Cases where mNGS indicates the presence of other major pathogenic bacteria.

### The mNGS detection of bronchoalveolar lavage fluid

The bronchoalveolar lavage fluid (BALF), from each participant, were collected from the lung segments indicated by the CT scan. Instill 20 mL of sterile saline into the affected lung segment and recover at least 10 mL of bronchoalveolar lavage fluid as a test sample.

DNA from each BALF sample (~1 mL) was extracted using the QIAamp DNA Micro Kit (Qiagen, Germany). Total RNA from 15 BALF samples was isolated with the QIAamp Viral RNA Kit (Qiagen, Germany), and rRNA was removed using the Ribo-Zero rRNA Removal Kit (Illumina, United States). cDNA was synthesized with reverse transcriptase and dNTPs (Thermo Fisher, United States). DNA and cDNA libraries were prepared using the QIAseq Ultralow Input Library Kit (Qiagen, Germany) and assessed for quality with a Qubit fluorometer and Agilent 2100 Bioanalyzer. The final libraries were sequenced on a NextSeq 550 platform (Illumina, United States) with 75-bp single-end reads, yielding approximately 20 million reads per library. The following bioinformatics analysis were conducted and reported by Vision Medicals (Guangzhou, China).

### Data collection

We utilized the electronic medical record system to gather comprehensive data, including general information, symptoms, signs, laboratory tests, pulmonary images, complications, treatments, scores, course of illness, and prognoses. The laboratory tests consisted of blood routine, inflammatory markers, blood coagulation, liver and kidney function, electrolyte levels, and enzyme levels. The scores used were the Sequential Organ Failure (SOFA) score, Acute Physiological and Chronic Health (APACHE II) score, and Glasgow Coma Scale (GCS) score. To measure the concentrations of Th1 cell-associated factors INF-γ, IL-2, and IL-12 in the remaining medical sera of different groups, we employed enzyme-linked immunosorbent assay (ELISA) kits from Thermo Fisher Scientific.

### Statistical analysis

We used R software (The R Foundation, http://www.r-project.org, version 3.6.1). We expressed the continuous variables as medians (interquartile range) and compared them using a *t*-test or a Mann–Whitney test, depending on whether they conformed to the normal distribution. We represented the classification variables with *n* (%) and compared them with the Fisher exact chi-square test. We analyzed potential predictors with multiple logistic regression analyses. We drew a scatter plot to explore the concentration of Th1 cell-related factors (INF-r, IL2, and IL12) in each group. *p* < 0.05 was considered statistically significant.

## Results

### Patient baseline characteristics

The CP and NP groups had similar sociodemographic characteristics and history of comorbidities. Among the patients in the CP group, 15.4% had a history of exposure to birds and poultry, and 23.1% had a history of alcohol consumption. The SP group had a higher number of comorbidities compared to the CP group (Table 1).

**Table 1:** Patient baseline characteristics.

	All (*n* = 52)			P (*n* = 39)		
Characteristics	NP (*n* = 39)	CP (*n* = 13)	*p*	CP (*n* = 13)	SP (*n* = 26)	*p*
Sex			0.87			0.482
Female	15 (38.5%)	6 (46.2%)		6 (46.2%)	8 (30.8%)	
Male	24 (61.5%)	7 (53.8%)		7 (53.8%)	18 (69.2%)	
Age	61.0 (53.0, 67.5%)	63.0 (55.0, 65.0%)	0.983	63.0 (55.0, 65.0)	60.5 (54.2, 70.0)	0.612
Exposure	1 (2.6%)	2 (15.4%)	0.151	2 (15.4%)	5 (19.2%)	1
Smoking	16 (41%)	4 (30.8%)	0.742	4 (30.8%)	13 (50%)	0.424
Drinking	5 (12.8%)	3 (23.1%)	0.396	3 (23.1%)	11 (42.3%)	0.304
Any comorbidity	18 (46.2%)	6 (46.2%)	1	6 (46.2%)	16 (61.5%)	0.568
Pulmonary disease	11 (28.2%)	2 (15.4%)	0.475	2 (15.4%)	2 (7.7%)	0.589
Cardiovascular disease	12 (30.8%)	3 (23.1%)	0.733	3 (23.1%)	13 (50%)	0.205
Cerebrovascular disease	0 (0%)	0 (0%)	1	0 (0%)	3 (11.5%)	0.538
Diabetes	2 (5.1%)	2 (15.4%)	0.257	2 (15.4%)	6 (23.1%)	0.694
Chronic kidney disease	1 (2.6%)	0 (0%)	1	0 (0%)	1 (3.8%)	1
Chronic liver disease	0 (0%)	1 (7.7%)	0.25	1 (7.7%)	6 (23.1%)	0.388
Malignancy	0 (0%)	0 (0%)	1	0 (0%)	4 (15.4%)	0.281

### Clinical symptoms and physical signs

All patients in the CP group had a fever, with a mean temperature of 39.6°C. The duration of fever in the CP group was longer than in the NP group, while cough and sputum were similar between the two groups. Patients in the CP and SP groups were more likely to experience dyspnea and extrapulmonary symptoms such as fatigue, muscle soreness, and diarrhea compared to the NP group. Additionally, the CP group had a faster respiratory rate, a relatively slow pulse, and lower blood pressure compared to the NP group. The SP group had a more rapid respiratory rate, lower blood pressure, and lower oxygen saturation than the CP group (Table 2). The etiology data of NP group participants are listed in Table 3.

**Table 2:** Clinical symptoms and signs.

	All (*n* = 52)			P (*n* = 39)		
Variables	NP (*n* = 39)	CP (*n* = 13)	*p*	CP (*n* = 13)	SP (*n* = 26)	*p*
Symptoms						
Fever	16 (41%)	13 (100%)	<0.001	13 (100%)	26 (100%)	1
Highest temperature, °C	36.9 (36.6, 39.0)	39.6 (39.2, 40.0)	<0.001	39.6 (39.2, 40.0)	39.8 (39.0, 40.0)	0.752
Fever days	1.0 (0.0, 7.0)	11.0 (9.0, 15.2)	<0.001	11.0 (9.0, 15.2)	11.0 (8.0, 15.2)	0.659
Cough	34 (87.2%)	9 (69.2%)	0.203	9 (69.2%)	23 (88.5%)	0.194
Sputum production	30 (76.9%)	8 (61.5%)	0.3	8 (61.5%)	24 (92.3%)	0.03
Chest pain	7 (17.9%)	0 (0%)	0.171	0 (0%)	3 (11.5%)	0.538
Headache	2 (5.1%)	3 (23.1%)	0.093	3 (23.1%)	5 (19.2%)	1
Fatigue	10 (25.6%)	9 (69.2%)	0.008	9 (69.2%)	22 (84.6%)	0.402
Myalgia	1 (2.6%)	6 (46.2%)	<0.001	6 (46.2%)	16 (61.5%)	0.568
Diarrhea	0 (0%)	2 (15.4%)	0.059	2 (15.4%)	7 (26.9%)	0.689
Dyspnea	25 (64.1%)	11 (84.6%)	0.298	11 (84.6%)	26 (100%)	0.105
Signs						
Fastest respiratory rate	21.0 (21.0, 22.0)	22.0 (21.0, 23.0)	0.03	22.0 (21.0, 23.0)	32.5 (24.2, 35.8)	<0.001
Fastest heart rate	95.0 (89.0, 105.0)	108.0 (94.0, 115.0)	0.06	108.0 (94.0, 115.0)	115.0 (108.0, 122.0)	0.029
Lowest systolic BP	115.0 (104.0, 122.0)	102.0 (95.0, 110.0)	0.01	102.0 (95.0, 110.0)	94.0 (89.2, 100.0)	0.052
Lowest diastolic BP	74.0 (69.0, 78.0)	65.0 (63.0, 68.0)	0.006	65.0 (63.0, 68.0)	58.0 (52.0, 63.8)	0.003
Lowest oxygen saturation	95.0 (92.0, 96.0)	94.0 (92.0, 96.0)	0.371	93.5 (91.5, 96.0)	90.0 (88.0, 91.0)	0.001
Change of consciousness	0 (0%)	0 (0%)	1	0 (0%)	7 (26.9%)	0.073
Pulmonary auscultation			0.042			0.06
Moist rate	27 (69.2%)	5 (38.5%)		5 (38.5%)	18 (69.2%)	

**Table 3:** The etiology of NP group pneumonia.

Etiology	*n* = 39 (100%)
Mycoplasma	5 (12.82%)
*Streptococcus pneumoniae*	10 (25.64%)
*Stenotrophomonas maltophilia*	2 (5.13%)
Pneumocystis	1 (2.56%)
Monilia albican	4 (10.26%)
Haemophilus influenzae	7 (17.95%)
Respiratory syncytial virus	3 (7.69%)
*Escherichia coli*	2 (5.13%)
*Staphyloccocus aureus*	2 (5.13%)
Legionella	1 (2.56%)
Influenza virus	2 (5.13%)

### Laboratory test results

Compared to the NP group, the CP group showed a decrease in average blood lymphocytes and Hb levels. However, there was no significant difference in platelet, leukocyte, and neutrophil levels. The SP group had lower lymphocyte and platelet levels, and their Hb levels fell to the diagnostic criteria for anemia. Additionally, the inflammatory indicator C-reactive protein (CRP) increased significantly in both the CP and SP groups. The CP group also showed a decrease in liver function, with a decrease in albumin (ALB) levels and an increase in alanine aminotransferase (ALT) and aspartate aminotransferase (AST) levels. There was also an increase in the myocardial enzyme lactate dehydrogenase (LDH). In comparison, the SP group showed a significant increase in ALT, AST, and LDH levels, as well as an increase in myoglobin levels (Table 4).

**Table 4:** Laboratory test results.

	All (*n* = 52)			P (*n* = 39)		
Variables	NP (*n* = 39)	CP (*n* = 13)	*p*	CP (*n* = 13)	SP (*n* = 26)	*p*
WBC, ×10^9^/L	8.1 (6.9, 12.9)	8.6 (5.5, 9.8)	0.619	8.6 (5.5, 9.8)	7.8 (4.8, 12.4)	0.952
Neutrophil, ×10^9^/L	6.4 (4.5, 10.6)	7.0 (4.0, 9.0)	0.992	7.0 (4.0, 9.0)	6.6 (3.6, 11.8)	0.893
Lymphocyte, ×10^9^/L	1.1 (0.8, 1.8)	0.8 (0.6, 1.0)	0.057	0.8 (0.6, 1.0)	0.4 (0.3, 0.7)	0.033
Hb, g/L	130.0 (119.5, 140.5)	110.0 (99.0, 127.0)	0.026	110.0 (99.0, 127.0)	94.5 (83.2, 106.2)	0.006
Platelet, ×10^9^/L	254.0 (204.5, 319.0)	247.0 (138.0, 300.5)	0.411	247.0 (138.0, 300.5)	125.0 (80.0, 224.5)	0.028
CRP, mg/L	47.5 (0.0, 119.4)	115.3 (50.8, 224.2)	0.019	115.3 (50.8, 224.2)	245.5 (156.5, 275.8)	0.102
ESR, mm/h	102.0 (36.5, 120.0)	120.0 (102.2, 120.0)	0.052	120.0 (102.2, 120.0)	104.5 (50.8, 120.0)	0.205
PCT, ng/mL	0.0 (0.0, 0.1)	0.2 (0.1, 0.3)	0.009	0.2 (0.1, 0.3)	1.6 (0.8, 6.4)	0.001
Fibrinogen, g/L	5.2 (3.3, 6.7)	6.4 (5.2, 9.0)	0.049	6.4 (5.2, 9.0)	6.8 (3.9, 7.3)	0.375
D-dimer, mg/L	0.9 (0.3, 1.6)	1.4 (1.0, 1.8)	0.168	1.4 (1.0, 1.8)	7.0 (2.1, 13.4)	<0.001
ALB, g/L	34.5 (30.6, 38.2)	28.2 (23.5, 33.1)	0.014	28.2 (23.5, 33.1)	24.3 (22.2, 28.3)	0.118
ALT, U/L	27.0 (14.0, 53.0)	50.0 (36.0, 130.0)	0.016	50.0 (36.0, 130.0)	109.0 (73.0, 293.0)	0.065
AST, U/L	31.0 (18.0, 52.5)	90.0 (55.0, 200.0)	0.003	90.0 (55.0, 200.0)	184.0 (113.0, 393.0)	0.045
TB, μmol/L	9.6 (7.3, 11.3)	13.4 (8.7, 18.0)	0.128	13.4 (8.7, 18.0)	18.7 (13.2, 24.2)	0.043
BUN, mmol/L	5.0 (3.7, 6.3)	5.6 (3.7, 6.1)	1	5.6 (3.7, 6.1)	7.7 (5.1, 13.7)	0.015
Creatinine, μmol/L	69.0 (56.0, 80.0)	65.0 (60.0, 80.0)	0.792	65.0 (60.0, 80.0)	90.5 (65.8, 126.8)	0.121
Potassium, mmol/L	4.0 (3.7, 4.4)	3.9 (3.6, 4.2)	0.482	3.9 (3.6, 4.2)	3.9 (3.5, 4.6)	0.623
Sodium, mmol/L	140.3 (137.8, 141.4)	138.2 (135.5, 141.8)	0.294	138.2 (135.5, 141.8)	140.5 (135.0, 143.5)	0.561
CK, U/L	103.5 (63.2, 186.8)	77.5 (68.8, 125.8)	0.68	77.5 (68.8, 125.8)	298.0 (127.8, 578.5)	0.013
CKMB, U/L	18.0 (13.2, 22.8)	17.5 (14.8, 19.2)	0.763	17.5 (14.8, 19.2)	23.0 (18.2, 37.2)	0.038
LDH, U/L	222.0 (187.2, 276.8)	314.5 (250.5, 375.5)	0.023	314.5 (250.5, 375.5)	473.5 (375.0, 885.8)	<0.001
Myoglobin, ng/mL	49.0 (34.1, 106.2)	63.4 (42.0, 104.0)	0.299	63.4 (42.0, 104.0)	178.8 (96.3, 344.9)	0.002

### Pulmonary CT images changes

**Figure 2:**
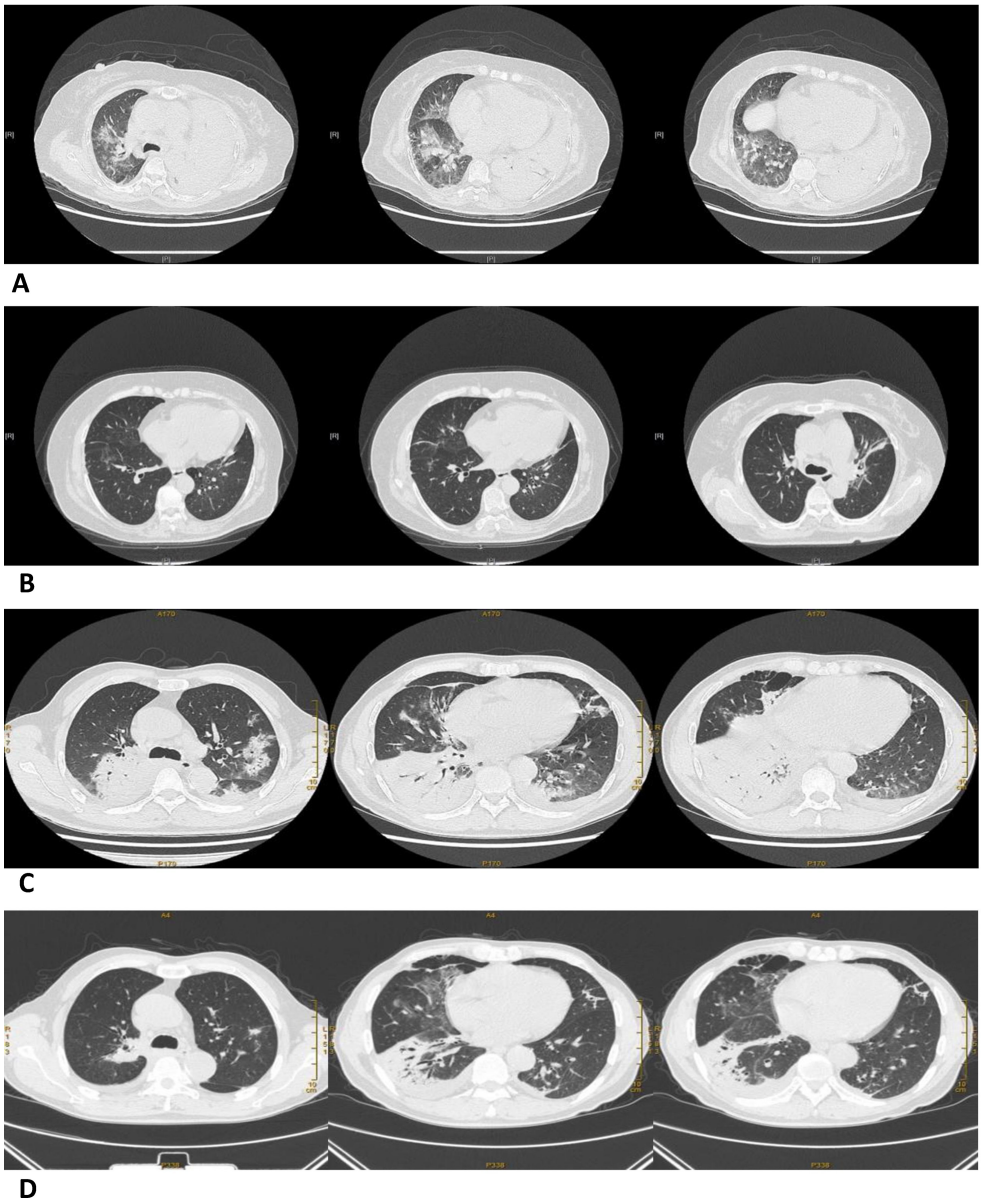
Pulmonary CT images before and after treatment in a severe and a common *Chlamydia psittaci* pneumonia patient. **(A)** Large consolidation of the left lung and patchy exudation shadow of the right lung before treatment (07/02/2021). **(B)** The area of consolidation and patchy exudation shadow significantly reduced after treatment (01/03/2021). Pulmonary CT images before and after treatment in a common *Chlamydia psittaci* pneumonia patient. **(C)** Large areas of exudation and consolidation in the right upper lobe and right lower lobe, bronchial inflation signs, and minor pleural effusion before treatment (18/09/2020). **(D)** Lesion clearly absorbed after treatment (29/09/2020).

The most common pulmonary CT features in the P group were consolidation and exudation, which are similar to other cases of community-acquired pneumonia (CAP). However, nodules and cavities were not present in any of the cases. Furthermore, the rate of *Chlamydia psittaci* pneumonia complicated with pleural effusion increased as the condition worsened. In the SP group, there was a higher likelihood of involvement in multiple lobes compared to the CP group, with 65.4% of patients having 4–5 lobes affected (Table 5 and Figure 2).

**Table 5:** Pulmonary CT images changes.

	All (*n* = 52)			P (*n* = 39)		
Variables	NP (*n* = 39)	CP (*n* = 13)	*p*	CP (*n* = 13)	SP (*n* = 26)	*p*
Diseased lobes ≤3	22 (56.4%)	9 (69.2%)	0.624	9 (69.2%)	9 (34.6%)	0.088
Diseased lobes >3	17 (43.6%)	4 (30.8%)	0.624	4 (30.8%)	17 (65.4%)	0.088
Double upper lobes	27 (69.2%)	11 (84.6%)	0.472	11 (84.6%)	23 (88.5%)	1
Double middle and lower lobes	36 (92.3%)	13 (100%)	0.564	13 (100%)	25 (96.2%)	1
Range of lung lesions	17.0 (9.0, 26.0)	21.0 (13.0, 31.0)	0.105	21.0 (13.0, 31.0)	38.0 (27.0, 59.5)	0.003
Consolidation	24 (61.5%)	11 (84.6%)	0.178	11 (84.6%)	22 (84.6%)	1
Exudation	36 (92.3%)	11 (84.6%)	0.589	11 (84.6%)	21 (80.8%)	1
Stripe	31 (79.5%)	3 (23.1%)	<0.001	3 (23.1%)	8 (30.8%)	0.719
Nodules	21 (53.8%)	0 (0%)	0.002	0 (0%)	1 (3.8%)	1
Inanition	1 (2.6%)	0 (0%)	1	0 (0%)	0 (0%)	1
Pleural effusion	11 (28.2%)	5 (38.5%)	0.506	5 (38.5%)	18 (69.2%)	0.135

### Complications and treatments

Compared to the CP group, all patients in the SP group were admitted to the intensive care unit (ICU). Additionally, 80.8% of these patients were diagnosed with acute respiratory distress syndrome (ARDS). The use of respiratory support therapies, including high-flow oxygen, noninvasive assisted ventilation, invasive assisted ventilation, and extracorporeal membrane oxygenation (ECMO), significantly increased in the SP group. Furthermore, there was a higher frequency of treatment with glucocorticoids, albumin, and liver protection therapy in the SP group (Table 6).

**Table 6:** Complications and treatments.

	All (*n* = 52)			P (*n* = 39)		
Variables	NP (*n* = 39)	CP (*n* = 13)	*p*	CP (*n* = 13)	SP (*n* = 26)	*p*
Complications						
ICU	0 (0%)	0 (0%)	1	0 (0%)	26 (100%)	<0.001
ARDS	4 (10.3%)	0 (0%)	0.561	0 (0%)	21 (80.8%)	<0.001
Shock	0 (0%)	1 (7.7%)	0.25	1 (7.7%)	4 (15.4%)	0.648
Acute kidney injury	0 (0%)	2 (15.4%)	0.059	2 (15.4%)	3 (11.5%)	1
Treatment						
Anti-infection	38 (97.4%)	13 (100%)	1	13 (100%)	26 (100%)	1
Hormones	4 (10.3%)	3 (23.1%)	0.347	3 (23.1%)	12 (46.2%)	0.295
Immunoglobulin	0 (0%)	0 (0%)	1	0 (0%)	3 (11.5%)	0.538
Human albumin	6 (15.4%)	4 (30.8%)	0.244	4 (30.8%)	23 (88.5%)	<0.001
Hepatoprotective drugs	6 (15.4%)	8 (61.5%)	0.003	8 (61.5%)	21 (80.8%)	0.253
Oxygen support						
Nasal cannula	25 (64.1%)	11 (84.6%)	0.298	11 (84.6%)	20 (76.9%)	0.694
High flow nasal cannula	1 (2.6%)	1 (7.7%)	0.441	1 (7.7%)	11 (42.3%)	0.034
Noninvasive ventilation	3 (7.7%)	0 (0%)	0.564	0 (0%)	19 (73.1%)	<0.001
Invasive mechanical ventilation	0 (0%)	0 (0%)	1	0 (0%)	7 (26.9%)	0.073
ECMO	0 (0%)	0 (0%)	1	0 (0%)	1 (3.8%)	1

### Score, duration, and prognosis

The SP group had significantly higher SOFA and APACHE II scores compared to the CP group, and their hospitalization and ICU admission lengths were longer. However, the course of disease was shorter in the SP group compared to the CP group (Table 7).

**Table 7:** Score, duration, and prognosis.

	All (*n* = 52)			P (*n* = 39)		
Variables	NP (*n* = 39)	CP (*n* = 13)	*p*	CP (*n* = 13)	SP (*n* = 26)	*p*
SOFA scores	15.0 (15.0, 16.0)	16.0 (15.0, 18.0)	0.031	16.0 (15.0, 18.0)	19.0 (17.0, 20.8)	0.02
APACHE II scores	6.0 (4.0, 9.0)	10.0 (8.0, 11.0)	0.004	10.0 (8.0, 11.0)	18.5 (13.2, 25.0)	<0.001
GCS scores	15.0 (15.0, 15.0)	15.0 (15.0, 15.0)	0.234	15.0 (15.0, 15.0)	14.0 (9.8, 15.0)	0.002
Course of disease	10.0 (6.5, 25.0)	10.0 (7.0, 15.0)	0.941	10.0 (7.0, 15.0)	7.5 (7.0, 19.0)	0.94
Days in hospital	7.0 (5.0, 8.5)	10.0 (7.0, 12.0)	0.027	10.0 (7.0, 12.0)	15.0 (12.5, 19.0)	0.001
ICU time	0.0 (0.0, 0.0)	0.0 (0.0, 0.0)	1	0.0 (0.0, 0.0)	6.5 (5.0, 10.8)	<0.001
Prognosis			1			1
Discharge	38 (97.4%)	13 (100%)		13 (100%)	25 (96.2%)	
Death	1 (2.6%)	0 (0%)		0 (0%)	1 (3.8%)	

### Predictors of *Chlamydia psittaci* pneumonia

**Figure 3:**
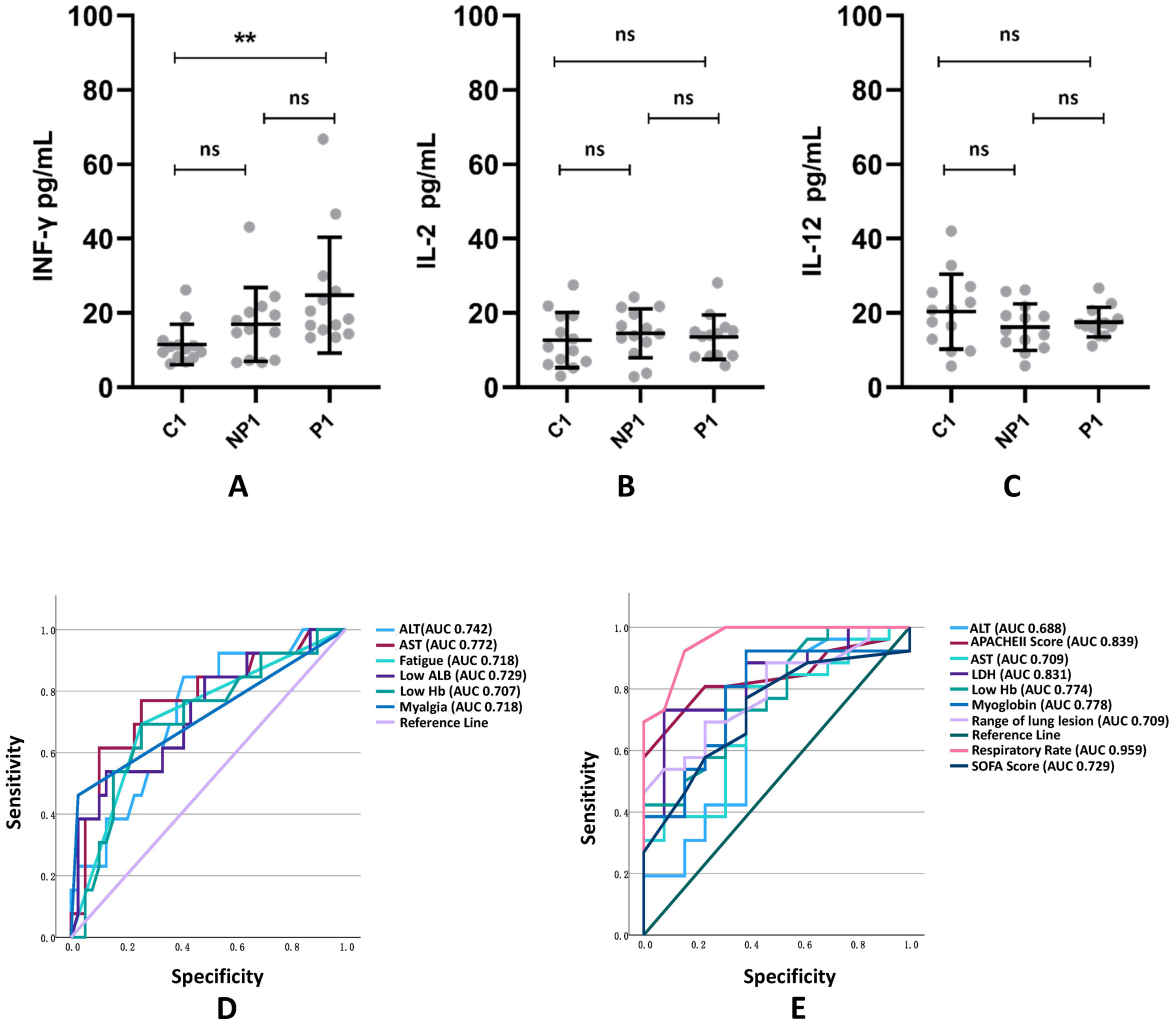
Comparison of Th1-related cytokines INF-γ **(A)**, IL-2 **(B)**, and IL-12 **(C)** among C1 healthy population group, NP1 non-psittacosis community-acquired pneumonia group, and P1 *Chlamydia psittaci pneumonia* group. The ROC curve and related AUC of CP pneumonia **(D)** and severe CP pneumonia **(E)** predictors are exhibited. ^**^p < 0.01; ns, the difference was not statistically significant.

Multiple logistic regression analysis and receiver operating characteristic (ROC) curve found that fatigue, myalgia, low hemoglobin levels, low albumin levels, elevated ALT levels, and elevated AST levels are all predictors for *Chlamydia psittaci* pneumonia (Table 8), with excellent predictive performance (Figure 3D). The area under the curve (AUC) of each predictor.

**Table 8:** Predictors of *Chlamydia psittaci* pneumonia.

Variables	OR (95% CI)	*p*
Fatigue	6.38 (1.59–25.59)	0.009
Myalgia	32.70 (3.35–319.41)	0.003
Hb, g/dL	0.96 (0.93–1.00)	0.032
ALB, g/L	0.84 (0.74–0.96)	0.011
ALT, U/L	1.01 (1.00–1.02)	0.015
AST, U/L	1.01 (1.00–1.03)	0.007

### Predictors of severe *Chlamydia psittaci* pneumonia

Multiple logistic regression analysis (Table 9) and ROC curve (Figure 3E) revealed that fast respiratory rates, low Hb, high LDH, significant involvement of multiple lobes, high Sequential Organ Failure scores, and high Acute Physiological and Chronic Health scores were predictors for severe *Chlamydia psittaci* pneumonia.

**Table 9:** Predictors of severe *Chlamydia psittaci* pneumonia.

Variables	OR (95% CI)	*p*
Respiratory rates	2.5 (1.53–4.09)	<0.001
Hb, g/L	0.89 (0.82–0.97)	0.006
ALT, U/L	1.00 (1.00–1.01)	0.161
AST, U/L	1.01 (1.00–1.01)	0.103
LDH, U/L	1.01 (1.00–1.03)	0.019
Myoglobin, ng/mL	1.01 (1.00–1.02)	0.055
Range of lung lesions	1.08 (1.01–1.14)	0.015
SOFA scores	1.37 (1.01–1.85)	0.043
APACHE II scores	1.64 (1.16–2.33)	0.005

### Th1-related cytokines

Compared to the C1 and NP1 groups, the P1 group showed a significant increase in INF-γ levels. However, there was no significant difference in IL-2 and IL-12 levels among the groups (see Figure 3).

## Discussion

Concerning *Chlamydia psittaci* pneumonia, previous studies have suggested that its clinical manifestations are similar to other cases of community-acquired pneumonia (CAP) (19). However, due to the difficulty in isolating and identifying *Chlamydia psittaci* using traditional cultural methods, diagnosing the pathogen can be challenging (20, 21). Treatment with common drugs used for CAP, such as cephalosporins, penicillins, and other β-lactam antibiotics, is often ineffective. In some cases, patients may even develop critical conditions or die without timely and effective treatment (12). Therefore, early recognition of the infection is crucial for improving prognosis. In this study, current cases were diagnosed using mNGS, a massively parallel sequencing technique for determining nucleotide sequences (13, 14). The analysis of 39 *Chlamydia psittaci* patients using mNGS confirms the validity of this test for diagnosing the infection. However, mNGS is an expensive and unpopular method. As a result, it is important to distinguish this disease early on from other cases of CAP, promptly identify severe patients, and search for possible peripheral blood markers as urgent clinical tasks. Besides, real-time polymerase chain reaction (RT-PCR) is also one of the effective tests for CP pneumonia and this method which is more time-, money- and labor-saving, especially in the duration of CP pneumonia outbreak (22). However, when dealing with sporadic cases, mNGS has greater diagnostic value, as it enables the timely detection of nearly any lower respiratory tract pathogen.

Previous case reports and a few studies have summarized some features of *Chlamydia psittaci* pneumonia (23–25). However, this study is the first to compare the clinical features, laboratory tests, and imaging characteristics of *Chlamydia psittaci* pneumonia with those of non-psittacosis community-acquired pneumonia (CAP). Our findings indicate that the majority of cases with avian exposure were caused by *Chlamydia psittaci*. This bacterium primarily infects birds and poultry (12), and humans typically contract it through inhalation or contact with the secretions of sick birds and poultry (6, 26–29). Human-to-human transmission is rare (30). Previous studies have identified bird and poultry exposure as the main risk factor (3), highlighting the importance of collecting thorough medical histories. Additionally, 23.1% of the CP group had a history of chronic alcohol consumption, and nearly 7.7% had chronic liver disease. Further these individuals. In our study, 61.5% of the SP group had comorbidities, as *Chlamydia psittaci* often infects immunocompromised hosts (31). Therefore, we must remain vigilant for severe infections in patients with underlying diseases who are infected with *Chlamydia psittaci*.

According to previous case reports, *Chlamydia psittaci* pneumonia can affect multiple systems, including the respiratory, digestive, cardiovascular, blood, urinary, nervous, and skin barrier systems (11, 32–40). The clinical symptoms of *Chlamydia psittaci* pneumonia lack specificity and can vary in severity, with the potential to rapidly progress from asymptomatic infection to severe pneumonia with multiple organ failure (7–10, 12, 39). In our study, we observed that hyperthermia, dyspnea, and extrapulmonary manifestations such as fatigue, myalgia, and diarrhea were more common in the CP group compared to the NP group. We also found that CP pneumonia patients are more likely to display fatigue and myalgia. Therefore, patients with community-acquired pneumonia (CAP) who present with extrapulmonary symptoms of fatigue and myalgia should be monitored for possible *Chlamydia psittaci* infection. Interestingly, we did not observe any abnormal electrolyte levels in patients with psittacosis, which differs from *Legionella pneumophilia* pneumonia, which often presents with similar extrapulmonary symptoms (19, 41). In contrast, patients with *Legionella pneumophilia* pneumonia often have polyhyponatremia and low blood phosphorus levels (42). In terms of physical signs, we noted that significantly higher respiratory rate (tachypnea) in the CP group, with more pronounced abnormalities in the SP group. Our statistical analysis revealed that tachypnea is very likely to be associated with severe *Chlamydia psittaci* pneumonia. Additionally, we found that a high proportion of severe patients (66.7%) required admission to the intensive care unit (ICU), highlighting the importance of early recognition and effective treatment in improving outcomes for patients with *Chlamydia psittaci* pneumonia.

This study not only analyzed laboratory examinations such as blood routine, inflammatory indexes, liver function, enzymology, coagulation function, renal function, and electrolytes, but also explored the concentrations of Th1 cell-associated factors INF-γ, IL-2, and IL-12 in the remaining medical sera. We found that compared with the NP group, the lymphocytes and hemoglobin (Hb) levels in the blood routine of the CP group were decreased. In the SP group, these decreases were more significant, with most patients experiencing anemia and a significant decrease in platelet levels within the normal range. However, leukocyte and neutrophil levels did not increase, which is not consistent with the symptoms of hyperthermia. These abnormal changes in the blood routine may be related to the spread of *Chlamydia psittaci* in the blood and its involvement in the blood system (43). Statistical analysis found that low Hb is a predictor and risk factor for *Chlamydia psittaci* pneumonia and severe cases. When Hb levels decrease in patients with CAP, excluding obvious acute blood loss and chronic anemia, *Chlamydia psittaci* pneumonia should be considered, as the decline may be related to the severity of the infection. Inflammatory parameters such as CRP were found to be significantly increased in the CP group and even more so in the SP group, indicating a severe infection. The liver enzymes ALT and AST were also significantly elevated in the CP group and even more so in the SP group, which may be related to liver injury (7, 9). Clinical reports have shown elevated liver enzymes in patients with psittacosis (20, 44), and veterinary pathology studies have shown liver involvement in infected parrots (45). This suggests that the liver may be a relatively susceptible site for *Chlamydia psittaci* pneumonia. We also found reduced levels of albumin (ALB) in patients with *Chlamydia psittaci* pneumonia. In addition to liver damage, *Chlamydia psittaci* is a specialized intracellular pathogen that lacks many essential nutrients and requires amino acids from eukaryotic host cells (46), leading to hypoalbuminemia. Statistical analysis found that low Hb, high ALT, and high AST were predictors for *Chlamydia psittaci* pneumonia, and high ALT and AST were also predictors of severe cases. Therefore, when patients with CAP have impaired liver function, decreased Hb, and increased liver enzymes ALT and AST, they should be alert to the possibility of infection with *Chlamydia psittaci*. High ALT and high AST levels suggest a severe infection. Unlike previous studies that found abnormal levels of CK and BNP to be predictors for severe *Chlamydia psittaci* pneumonia (31), we found that LDH levels were increased in the CP group, especially in the SP group, and myoglobin levels were also increased. These could be associated with rhabdomyolysis and cardiac involvement (12, 47, 48). Statistical analysis found that high LDH levels were predictors and predictors for severe *Chlamydia psittaci* pneumonia, and high myoglobin levels were also predictors of severe cases. The increase in LDH and myoglobin levels may suggest a serious condition in patients with *Chlamydia psittaci* pneumonia. In contrast, there were no obvious abnormalities in coagulation function, renal function, and electrolytes in patients with *Chlamydia psittaci* pneumonia, which may serve as exclusionary diagnostic references and differentiation points. In this study, the Th1 cell-associated factor INF-γ was found to be elevated in the P1 group. Previous studies have shown that INF-γ can induce the iron deficiency pathway by upregulating the expression of divalent metal ion transporter 1 in monocyte macrophages and downregulating the transferrin receptor of infected cells and ferrous transporter in monocyte macrophages (49). This limits the uptake of iron, an essential element for chlamydia growth, and has an anti-chlamydia infection effect (50, 51). Therefore, the increase in INF-γ levels may be related to the condition and should be investigated in the future as a possible peripheral blood marker. Besides, another study also explored the immune reactions in *Chlamydia psittaci* pneumonia and they found psittacosis cases with pneumonia had higher levels of serum cytokines (G-CSF, IL-2, IL-6, IL-10, IL-18, IP-10, MCP-3, and TNF-α) than bronchitis cases (17). Among them, IL-6 contributes to both pneumonia pathogenesis and iron restriction during infection by increasing hepcidin that causes iron restriction in blood and traps the iron intracellularly (52). This effect may lead to the lower availability of iron for Hb synthesis thus leading to lower Hb levels but paradoxically promote the multiplication of intracellular pathogens like *Chlamydia psittaci* (53).

The CT findings in this study were generally consistent with those of previous studies (44, 54). The predominant imaging pattern of psittacosis was consolidation and exudative (55). Nodules and cavities were rare, which could be used as exclusionary diagnostic criteria and a differential point for *Chlamydia psittaci* pneumonia (55). Furthermore, we observed that psittacosis often involved multiple lobes and resulted in more pleural effusion, indicating a more severe disease. Statistical analysis revealed that extensive involvement of multiple lobes is a predictor and risk factor for severe *Chlamydia psittaci* pneumonia, which can aid in determining the severity of the disease. In the SP group, most patients also had acute respiratory distress syndrome (ARDS), which may be attributed to the fact that *Chlamydia psittaci* primarily affects the lungs. Additionally, some patients had multiple organ dysfunction and sepsis in the past (10), and severe cases can be life-threatening.

Anti-infective treatment for *Chlamydia psittaci* pneumonia is most effective with tetracyclines, followed by macrolides, and quinolones (56, 57). However, conventional CAP anti-infective drugs such as cephalosporins, penicillins, and other β-lactam antibiotics have poor treatment effects due to the intracellular nature of the pathogenic bacteria. In our study, patients with *Chlamydia psittaci* pneumonia were treated with the aforementioned anti-infective therapy and showed significant improvement, with most being discharged. Out of 39 patients, only one severe case resulted in death, accounting for 2.6%. In addition to anti-infective treatment, a comprehensive respiratory support regimen, glucocorticoids, albumin, and hepatoprotective treatment were also utilized for patients with *Chlamydia psittaci* pneumonia. The intensity and abundance of treatment increased with the severity of the condition. Previous studies have reported that fulminant *Chlamydia psittaci* pneumonia can lead to multiple organ failures, requiring additional treatments such as cardiopulmonary bypass and hemodialysis (12). Timely and effective drug treatment has shown to have a positive response in *Chlamydia psittaci* pneumonia cases (25, 37). Early diagnosis is crucial for prompt and effective treatment.

Patients in the SP group had higher SOFA and APACHE II scores compared to the CP group. According to our analysis, high SOFA and APACHE II scores were found to be predictors for severe *Chlamydia psittaci* pneumonia. These scores can serve as reference indexes to aid in determining the severity of *Chlamydia psittaci* pneumonia. The SP group had longer hospital stays and were more likely to be admitted to the ICU, indicating a heavier disease burden. However, the SP group had a shorter disease course compared to the CP group, suggesting a faster progression of severe psittacosis pneumonia.

In summary, this study identified predictors for *Chlamydia psittaci* pneumonia and severe cases, as well as explored potential peripheral blood biomarkers. These findings can aid in early detection of *Chlamydia psittaci* pneumonia, prompt identification of severe cases, and potentially lead to shorter disease courses and improved outcomes.

However, this study has some limitations. Firstly, it was a single-center study conducted in a tertiary-care teaching hospital, which may introduce bias. Secondly, it was a retrospective study and some patients were difficult to track. Lastly, the sample size was small and may not fully represent the clinical features of *Chlamydia psittaci* pneumonia. Future prospective studies with larger, multicenter samples should be conducted.

## Conclusion

Fatigue, myalgia, low Hb, low ALB, high ALT, and high AST are predictors of *Chlamydia psittaci* pneumonia. Fast respiratory rates, low Hb, high LDH, significant involvement of multiple lobes, high Sequential Organ Failure scores, and high Acute Physiological and Chronic Health scores are predictors of severe *Chlamydia psittaci* pneumonia. The increase of INF-γ may be related to the condition.

## Data Availability

The original contributions presented in the study are included in the article/supplementary material, further inquiries can be directed to the corresponding authors.
